# The Value of Community Building and Social Capital for Rural Communities Recovering from Disaster

**DOI:** 10.1007/s10900-026-01583-w

**Published:** 2026-05-24

**Authors:** Amber Anderson, Santina Contreras, Emineh Nercissian, Rita V. Burke

**Affiliations:** 1 Department of Population and Public Health Sciences, University of Southern California, Los Angeles, CA USA; 2 Department of Urban Planning and Spatial Analysis, University of Southern California, Los Angeles, CA USA

## Abstract

Community building and social capital are essential components of disaster resilience for rural communities. Understanding the specific needs of rural communities and how community building benefits them in times of disaster and recovery is paramount in developing disaster recovery plans for these unique communities. Researchers conducted two focus groups and various informant interviews with the goal of collecting a narrative overview of Happy Camp’s residents, recounting their experiences during and after the Slater Fire of 2020. Qualitative analysis of the transcripts identified themes regarding the importance of community mobilization, tribal support, social capital, community organization, external support and recovery, and rural community culture. This narrative overview calls to attention the strengths that rural communities have during disaster recovery, as well as opportunities to better support these communities. The findings from this study reinforce the importance of continuing to advance our understanding of the needs of rural communities in disasters.

**Supplementary Information:**

The online version contains supplementary material available at 10.1007/s10900-026-01583-w.

## Introduction

Rural communities often face numerous pre-existing vulnerabilities that can hinder disaster recovery, including higher levels of community isolation, financial insecurities, and larger proportions of elderly populations [1, 2]. They also typically experience low population density, limited resources, and communication challenges, all of which can act as barriers to disaster recovery [3]. These communities can have limited entry and exit points. This isolation can pose accessibility challenges during disasters and result in delayed access to aid [2]. Rural communities often face challenges accessing disaster funding and response, making increasing community self-reliance a key strategy for increasing disaster resilience [4].

One way to address these challenges and gaps in disaster response is through community-based disaster response [5]. Understanding how to support social bonds and capital in rural communities could prove valuable in building disaster resilience. Rural communities have shown a prevalence for higher levels of civic engagement and strong trust in their neighbors, leading to better disaster responses than those without [6]. The sense of community and social bonds in rural communities can lead to higher levels of self-reliance in disasters [1, 7]. Social capital refers to the relationships within a given community that contribute to coordination and collaboration [8]. Social capital has been demonstrated to be a crucial resource for rural communities recovering from disasters [6, 9]. Community building that leverages local problem-solving can often provide more tailored disaster response solutions [8]. The purpose of this study is to provide a qualitative illustration of the value of community building and social capital as part of building resilience in rural communities. This study also aims to provide valuable insights that responding agencies can leverage while helping support rural communities.

## Case Study Description

This study was conducted in Happy Camp, California, three years after the Slater Fire of September, 2020, occurred. Happy Camp is a small, rural community located in Siskiyou County, Northern California. In September of 2020, the Slater Fire swept through Happy Camp, burning over 160,000 acres, and much of the community’s housing [10]. Happy Camp has an estimated population of 905, with the two most common ethnic groups being white (483 residents) and American Indian and Alaskan Native (289 residents), followed by two or more non-Hispanic ethnicities (133 residents) [11]. Happy Camp is a relatively isolated community, with the closest incorporated city of Yreka being about seventy miles away. Due to its location, many residents rely on services provided within Happy Camp, with one of the main providers being the Happy Camp Community Center. This makes services provided by the tribe and community center integral to the health of the community. The U.S. Department of Agriculture’s Economic Research Service uses population thresholds and remoteness to categorize rural areas, assisting with policy-making, as these indicators also influence jobs and availability of services [12]. These communities range from level one to level four and are known as Frontier and Remote (FAR) Communities. Due to its remote geographic location and small population, Happy Camp is classified as a Level 4 Frontier and Remote (FAR) community [12].

A large part of the Happy Camp community is formed by the Karuk tribe. In 2015, the Karuk tribe reclaimed over 1,600 acres of their tribe’s aboriginal land in Siskiyou County (Karuk Tribe, 2015). Karuk Tribal Headquarters are located in Happy Camp, providing various health and wellness services to many of the residents of Happy Camp (Karuk Tribe, 2015). The tribe plays an integral role in the functioning of the overall community.

### Methods

This study received institutional review board approval (UP-21-01222) from the University of Southern California. Data for this study were collected between February and April of 2024 through a series of key informant interviews and focus groups. Happy Camp Community Action (HCCA) and Resilience Inclusion Support Education (RISE) Collaborative were collaborators on this qualitative study. HCCA is a non-profit focused on promoting the health of Happy Camp residents and the community at large. HCCA provides residents with numerous resources, including, but not limited to, Slater Fire recovery support, community food programming, parenting classes, and mental health outreach [13]. RISE Collaborative is a community-based long-term Slater Fire recovery group that provides various resources for survivors of the Slater Fire, all with the focus of supporting community resilience [10]. Prior to participation, all interviewees were provided with consent forms. Signed forms were brought back with those who chose to participate.

## Study Population and Recruitment

Eligibility for focus group participation was open to residents of Happy Camp who survived the Slater Fire. All participants were required to be legal adults at the time the study was conducted. Community partners shared the study details with interested participants and conducted the recruitment process. In addition to focus groups, this study also conducted interviews with key informants. Individuals chosen to participate in the interviews included both community members and organizations that played integral roles in the Slater Fire recovery. These individuals were also selected by community partners for participation in the study.

## Focus Groups and Key Informant Interviews

Focus groups and key informant interviews were conducted at the Happy Camp Community Center, with an experienced moderator, and were audio recorded for later analysis. A total of two focus groups were conducted with six and three participants, respectively. The focus groups averaged thirty-nine minutes. Roughly 67% of the focus group participants were females. Participants were an average age of 32.6. Community partners contributed to the development of a discussion guide to facilitate focus group discussions. Key informant interviews were conducted with participants from various organizations that provided disaster support for Happy Camp, including but not limited to immediate emergency services and long-term recovery assistance. These interviews averaged approximately forty minutes. Moderators used a discussion guide that was also developed in collaboration with Happy Camp community partners to help facilitate discussion. The main purpose of the focus groups was to gain the community’s perspectives on the strengths and challenges associated with the disaster recovery process in a rural community. Key informant interviews were conducted to gain a deeper understanding of recovery approaches implemented after the Slater Fire, as well as to determine which support was most beneficial to the community.

## Data Collection and Analysis

All key informant interviews and focus groups were audio recorded. Audio recordings were transcribed via *Otter.ai* and manually validated and corrected. Interview transcripts were then analyzed in ATLAS.ti, a qualitative data analysis and coding software package. Using a mixed coding approach, an initial codebook was developed. Additional codes were inductively added as data analysis progressed. One researcher conducted the initial coding independently. An additional researcher reviewed and calibrated the results. Conventional content analysis was used to assimilate the qualitative data. A set of codes was developed and the criteria for assigning a specific code to a block of text was systematically developed by the primary coder (AA.). The coding scheme was refined and expanded to reflect and incorporate emerging themes throughout the coding process.

## Results

Through the focus groups and interviews, participants shared their opinions and experiences surrounding the Slater Fire and the recovery process. From the analysis, six main themes emerged: (1) community mobilization, (2) tribal support, (3) social capital, (4) community organization, (5) external support and recovery, and (6) rural community culture. Detailed quotes to support the identified themes can be found in Table 1.

1. *Community Mobilization.* Participants emphasized the value of community mobilization, particularly in relation to community-led efforts following the Slater Fire. Members of the community highlighted how initiatives from within the community were integral in supporting people who were unable to evacuate, stating, “Our employees…that’s what they spent a whole week doing was just sneaking behind the cops to check on people, to take things to them, to get them air purifiers and take them boxes of food and water.” They also discussed how experiencing the Slater Fire and the internal community support that followed helped strengthen community ties, sharing, “It was nice to see the portions of town, [with] people who didn’t lose their houses, how much they pulled together…After going through something so bad, it made them feel connected to each other.” Quotes 1 to 7 in Table 1 are included to provide additional context for these points.
2. *Tribal Support.* Participants emphasized the critical role that the Karuk Tribe played in the recovery from the Slater Fire. The Karuk tribe serves as a pillar within Happy Camp, and their established community bonds and understanding of the community’s needs have made them an instrumental contributor to Happy Camp’s recovery. The tribe effectively supported the community through its recovery, while also navigating required coordination with external partners. This position was affirmed through many community members sharing their gratitude to the Tribe for their role in procuring trailers to house those in need, engaging with government organizations to obtain a federal disaster declaration, and general support of Happy Camp after the Slater Fire, stating, “We lived in a tent for a little bit. And then, you know, we wanted to come back home and put the kids back to school and [go] back to work….we couldn’t have done that without the help of the Tribe. And they were the ones helping everybody. You know, without them we would [have] all been out.” Another resident also stated, “I think that the tribe was huge. And what they were able to, to, I mean, I think they’re, they’re literally the only reason the town survived.” Quotes 8 through 13 in Table 1 further support these findings and continue to illustrate the value of the Karuk tribe’s support.
3. *Social Capital.* Participants recognized the importance of social capital in a small, rural community. One participant stated, “We have ancestors from that area locally, tribal, you know, it’s part of our heritage to grow up on here…and so, when thinking about that, yeah, like, there’s just so much connection between all of the communities.” Participants discussed how bonds within the community existed before the fire were beneficial during the recovery, stating, “A lot has changed around just the fire, but even before the fire, the reality is there’s just a sense of community that is just the most amazing thing that can’t be trained.” Quotes 14 to 17 in Table 1 illustrate these findings.
4. *Community Organization.* Participants explained how efforts to continue building community after the Slater Fire were an important part of recovery, stating, “You live within the resources that you have in the community. What’s the most prominent resource in rural communities?… It’s the people who are your neighbors from seven generations down.” The desire to rebuild the community from within was strong, but resources were strained, with one participant stating, “It was after the Slater Fire, it was kind of hard because the community came together, we did come together, but…there was not enough resources to really come down and be like, in this area for the people.” Quotes 18 to 20 in Table 1 further illustrate these findings.
5. *External Support and Recovery.* While participants shared the value and ease of accessibility of community-based support, they also shared numerous frustrations dealing with external organizations that provided support during the Slater Fire recovery. Many individuals shared that they felt like external resources provided very limited help and were removed prematurely, stating, “You know, it took the tribe to do a lot of stuff. Why wasn’t the county helping? Why wasn’t the state office of emergency [helping]? What were those groups that are supposed to be doing a lot of that stuff, FEMA? Why aren’t they down here and helping? And why is it [external organizations] only stay down here for so long? They should be here still.” Residents also felt that some organizations did not understand the importance of placing recovery efforts on rebuilding Happy Camp, but rather placed emphasis on relocation. One example of this sentiment that was shared:

They set up their Red Cross stuff out in Yreka. Initially, when the evacuation happened, then the lift happened 11 days later. They didn’t want families to return to Happy Camp, they actually told people who had lost their home, well, you don’t live in Happy Camp…you live in Yreka. And for people who are, rooted here and very connected, that was a huge blow to somebody who had just lost all their belongings. And it really pissed us off. It was so upsetting.

Quotes 21 to 25 in Table 1 are included to illustrate these points.

*6. Rural Community Culture*. Residents of Happy Camp expressed that, as a community, they share common values and experiences, which in turn shape their own community culture. Participants shared a sense of mistrust and trepidation working with people and organizations that were not local and did not have an understanding of the needs of the community, sharing statements like, “I think, in Happy Camp, when they hear the word ‘government’ [it] really turns a lot of people off for some reason…I don’t know why that is. But it does. I think you might find that in a lot of rural areas…I mean, I think the [assistance with] food…everybody [was] cool with that, [but]in the case management, when you have to ask them for more personal information. They’re very reluctant to give it.” In addition to mistrust of external organizations, residents felt strongly about not relocating outside of Happy Camp. An example of the social-place attachment was shared by a participant by noting, “We started hearing from the Red Cross people that hotels and Yreka were their solution. Yeah, like, that’s where we were all gonna live long-term. And it was like, I never even stayed at the hotel and Yreka when I was evacuated, like why would I want to live in Yreka?” Quotes 26 to 28 in Table 1 illustrate this point.

## Discussion

Participants shared examples of the various ways that pre-existing community ties aided in recovery from the Slater Fire, citing examples of broad community-led efforts and neighborly support. Participants clearly shared that they are their own most valuable resource, forged over generations of familial and community bonds. Literature supports the importance of these bonds in disaster recovery, emphasizing that existing social capital has been shown to influence how quickly communities can work together during and after disasters [9]. Increasing community participation in disaster recovery can help reduce the emergency phase of disasters and reduce disaster recovery challenges [14]. As such, supporting rural communities in forming these integral bonds can potentially influence their disaster resilience and ability to respond to disasters.

One of the most frequently expressed sentiments among the participants was the irrefutable role that the Karuk tribe played in the recovery from the Slater Fire. Community members valued the Tribe’s efforts to preserve and rebuild Happy Camp, as well as their role corresponding with broader government entities to secure needed funding for emergency trailers and the federal disaster declaration, which helped catalyze the influx of various other resources for the community. The First Nations Development Institute reports that 54% of American Indians and Alaska Natives reside in either rural or small towns [15]. Additionally, there is significant variation between tribes, each with its own cultural considerations and sovereignty [16]. The importance of supporting partnerships with tribes is well-documented and supported by FEMA’s Tribal Mitigation Policy [16]. The Slater Fire recovery provides a powerful example of how tribal engagement can be an essential part of disaster recovery in rural communities, highlighting the importance of partnering with tribal entities to better support rural communities recovering from disasters. Partnering with tribes throughout the entire disaster cycle allows for enhanced coordination, while also allowing tribes to help determine relevant community health priorities and define appropriate responses to disasters [17].

Survivors of the Slater Fire also shared frustrations regarding their interactions with outside agencies during the recovery process. Participants shared that many were forced to evacuate to Yreka during the Slater Fire, making Yreka the epicenter for recovery resources. Residents shared that when they were able to return to Happy Camp, many external resources did not relocate or expand to Happy Camp. Many participants shared that they felt they were being pushed out of Happy Camp due to the availability of resources in Yreka and expressed mistrust of external agencies. Many rural residents experience a high level of social-place attachment, where they feel connected to their location through cultural and community ties [18]. Social-place attachments occur in rural communities when residents’ connections to family, neighbors, and their location can help shape how individuals define themselves, creating a personal bond between residents and their location [19]. This concept emphasizes the importance of providing local recovery resources whenever possible. Additionally, these families have lived in these communities for multiple generations, and the combination of family networks and local community expertise contributes to the high social capital of many rural communities, which can increase their resilience in disasters [20]. It is clear through discussions with residents that Happy Camp has its own culture that needs to be understood, valued, and respected in order to properly assist the community. Agencies supporting rural communities in disaster recovery can benefit from investing the time and effort to understand the specific needs of these communities and approach them with humility. FEMA highlights that building trust within rural communities enhances the effectiveness of making changes [21]. By supporting and leveraging the social bonds that rural communities have, in tandem with addressing their unique cultural needs, supporting agencies can more effectively aid them through the disaster recovery process.

One limitation of this study is that the results were derived from qualitative data collected from focus groups and interviews with a sample of the Happy Camp population, and may not be representative of all members of the Happy Camp community. This study may have unintentionally influenced how participants responded or failed to address relevant topics, potentially introducing biases. The strength of this study lies in its robust qualitative narrative analysis, which contributes to the existing literature on rural experiences with disasters. The study stresses the importance of community mobilization as a key element in rural disaster recovery. This illuminates the need to continue seeking ways to support rural communities’ ability to build resilience through social capital.

**Table 1:** Themes and quotes from focus group and key informant interview participants sharing their experiences with the Slater Fire and proceeding recovery*

Theme	Subtheme	Quote Number	Quote
Community Mobilization	Community-led efforts	1	The only people that have really been supportive I feel like was the tribe and this community center.
		2	It was also nice to see, like, the portions of town, the people that didn't lose their houses, like how much they pulled together to help the people who did me, you know, like donating stuff to the center, you know, and helping coordinate things and working on, you know, fire recovery and stuff like that…After going through something so bad, it made them feel connected to each other.
		3	Our employees, in housing that we had that were still in town, essentially, that's what they spent a whole week doing was just sneaking around behind the cops, to check on people to take people things to get them air purifiers to take them boxes of food and water.
		4	I think by having the community center here, it really became the community hub for resources and support…And what happens is they become the community hub, that's a safe place for supports and resources. They're not government. They're not you know, they're they're just a community based place where you can come in for support, but you also can come in and donate your time, like, you know, the the debt that's out there and helping, and it's a place where it's very community focused.
		5	What the community center has done is really become that large magnet that says, Come if you feel comfortable, okay, people come, you know, and then let's, and then we'll listen to their needs.
		6	But I guess is that community to driving force, or kind of recovery process, or actually think the fire brought the community closer together.
		7	And so it's like when things need done, we really pull together and community pulls together for the kids, they do a lot of fundraising to do things they gotta do, and everybody's like, donates to it, you know, we really work together to keep the community functioning as much as we can.
	Tribal Support	8	I think the tribe helped out a lot of people bringing the trailers and trailers for temporary housing
		9	I'm very thankful for the tribe because, you know, I witnessed it and in McKinney that didn't have the tribe as much. It was more people that helped during that fire to get like supplies basics, you know, toothpaste, you know, toilet paper, that basics to live off up and the tribe helped here so much. Without them, I don't think we would be even where we are.
		10	The only people that have really been supportive I feel like was the tribe and this community center. Yeah, the Karuk tribe housed us for 16 months we're still in tribal, they’re still housing us.
		11	We lived in a tent for a little bit. And then, you know, we wanted to come back home and put the kids back to school and back to work, you know, so we couldn't have done that without the help of the tribe. And they were the ones helping everybody. You know, without them we would all been out.
		12	I think that the tribe was huge. And what they were able to, to, I mean, I think they're, they're literally the only reason the town survived. Because the fear was, if they don't come home, they don't shop at the store, they don't use the post office, they don't put their kids in school towns gonna just disappear, you know, and disintegrate.
		13	There was one other place where the tribe was key. And that was in getting the Presidential the federal disaster declaration, because it was denied initially
Social Capital	Bonding Social Capital	14	There's almost more accountability between people within the community because everybody knows everybody. Pretty much everybody knows everybody's problems, issues, whatever is going through the community. So it's almost more accountability personal accountability.
		15	It's very close knit, you know, it's real small town, everybody knows everybody, everybody thinks they know each other's business. So there's a lot of overlap like that, which, which I say it could be good and bad, it's really good when something negative happens, because you can feel the town, you know, pull together and support each other.
		16	It's just such an incredibly beautiful, amazing community that has such a sense of community, a lot has changed. A lot has changed around just the fire, but then even before the fire, that the reality is there's just the sense of community that that is just the most amazing thing that's that can't be trained.
		17	We have ancestors from that area locally, tribal, you know, it's part of our heritage to grow up on here…and so, when thinking about that, yeah, like, there's just so much connection between all of the communities.
Community Organization	Community Building	18	You live within the resources that you have in the community. And what's the strongest, What's the most prominent resource in that in that rural communities?...It's the people who are your neighbors from seven generations down
		19	We offered a support group here. But at first started out really just informal, like, Hey, get out of your house and come down and do craft night with us or play games or whatever you wanted. And then it turned more into like a steady stream of people who came to craft night, there was a, there was a handful, that about 50%, in the beginning of people who lost their houses, and were just, you know, they didn't get the people who this was their hobbies beforehand, didn't have access to any of that stuff. And so that really helped people.
		20	It was after the Slater Fire, it was kind of hard because the community came together, we did come together, but then you also, you know, there was not enough resources to really come down and be like, in this area for the people.
External Support and Recovery		21	You know, it took the tribe to do a lot of stuff. Why wasn't the county helping? Why wasn't the state office of emergency? What were those groups that are supposed to be doing a lot of that stuff, FEMA? Why aren’t they down here and helping? And why is it only stay down here for so long? They should be here still.
		22	And then you turn around expecting you know, the county and stuff to help you out because you just want to be this big tragedy and really they're sitting there rooting for you to lose even more. Yeah, that's what it feels like.
		23	They set up their Red Cross stuff out in Yreka. Initially, when the evacuation happened, then then the lift happened to 11 days later. They didn't want families to return to Happy Camp, they actually told people who had lost their home. Well, you don't live in Happy Camp and you want more, you live in Yreka. And for people who are like, rooted here and very connected, that was a huge blow to somebody who had just lost all their belongings. And it felt really, it pissed us off. It was so upsetting.
		24	That was probably the hardest part to swallow was the idea that, you know, when Red Cross said that to them, you know, you don't live here, just go in and enroll your kid in another school. They can make new friends. That was pretty the hardest part because I have kids in that school. And that would be really tragic to just hear that like, oh, all of a sudden your kids to go to a Yreka elementary school instead of the one they've only ever known.
		25	FEMA is a huge entity, right. And they had a really very dismissive attitude about Happy Camp sort of, you know, fire and police, like personnel that were here they were, you know, wanting to starve people out, because they're not supposed to be here
Rural Community Culture		26	We started hearing from the Red Cross people that hotels and Yreka were their solution. Yeah, like, that's where we were all gonna live long term. And it was like I never even stayed at the hotel and Yreka when I was evacuated, like why would I want to live in Yreka and commute to Happy Camp.
		27	The other disaster case manager that was with me from NVCSS lived in Redding, okay. She did not get the same reception I got because people know me…So they were more comfortable coming to talk to me. Whereas they would request not to talk to her and come to me…So I think if they can actually hire people around the area of they won't be better.
		28	I think, in Happy Camp, when they hear the word government really turns a lot of people off for some reason. Okay. You know, I don't know why that is. But it does. I think you might find that in a lot rural areas…I mean, I think the food was, everybody's cool with that, like, in the case management, when you have to ask them for more personal information. They're very reluctant to give it.

* Quote numbers are used for organization only and do not correspond to individual participants

## Supplementary Information

Below is the link to the electronic supplementary material.

Supplementary Material 1.

## Data Availability

Data are not publicly available due to privacy restrictions but are available from the corresponding author on reasonable request.

## References

[1] Cutter, S. L., Ash, K. D., & Emrich, C. T. (2016). Urban–Rural Differences in Disaster Resilience. *Annals of the American Association of Geographers*, *106*(6), 1236–1252. 10.1080/24694452.2016.1194740

[2] García, I. (2024). Beyond Urban-Centered Responses: Overcoming Challenges to Build Disaster Resilience and Long-Term Sustainability in Rural Areas. *Sustainability*, *16*(11), 4373. 10.3390/su16114373

[3] Rural Health Information Hub (n.d.). *Rural emergency preparedness and response*. Retrieved from https://www.ruralhealthinfo.org/topics/emergency-preparedness-and-response

[4] Kapucu, N., & Rivera, F. (2020, December 15). *Rural Resilience: Disaster Preparedness for Communities off the Beathen Path*. Natural Hazards Center. https://hazards.colorado.edu/news/research-counts/rural-resilience-disaster-preparedness-for-communities-off-the-beaten-path

[5] Darab, S., Hartman, Y., & Pittaway, E. (2021). Building Community Resilience: Lessons from Flood-affected Residents in a Regional Australian Town. *Sage Journals*, *2*(4). 10.1177/2516602620981553

[6] Choo, M., & Yoon, D. K. (2022). Examining the effects of the local communities’ social capital on disaster response capacity in Seoul, South Korea. *International Journal of Disaster Risk Reduction*, *75*, 102973. 10.1016/J.IJDRR.2022.102973

[7] Jerolleman, A. (2020). Challenges of Post-Disaster Recovery in Rural Areas. In W. Sprigg, S. Lakshmi Steinberg, & S. Laska (Eds.), *Louisiana’s Response to Extreme Weather, A Coastal State’s Adaptation Challenges and Successes*. SpringerOpen. 10.1007/978-3-030-27205-0

[8] Winkler, M., Wallerstein, I., & Hyde, C. (2022). Improving Health Through Community Organizing and Community Building. In M. Minkler, & P. Wakimoto (Eds.), *Community Organizing and Community Building for Health and Social Equity* (4th ed., pp. 35–52). Rutgers University Prep.

[9] Panday, S., Rushton, S., Karki, J., Balen, J., & Barnes, A. (2021). The role of social capital in disaster resilience in remote communities after the 2015 Nepal earthquake. *International Journal of Disaster Risk Reduction*, *55*, 102112. 10.1016/J.IJDRR.2021.102112

[10] *2020 Slater Fire*. (2024). RISE Collaborative. https://www.happycampstrong.org/slater-fire-information

[11] U.S. Census Bureau (2020). *Happy Camp CDP, California Profile.* https://data.census.gov/table/DECENNIALPL2020.P1?g=160XX00US0632030

[12] Economic Research Service (n.d.) *Documentation: Frontier and Remote Area Code* [Dataset]. U.S. Department of Agriculture. Retrieved from https://www.ers.usda.gov/data-products/frontier-and-remote-area-codes/documentation/

[13] *Happy Camp Community Center, About Us*. (2016). Happy Camp Community Center. http://www.happycampcc.org/about.html

[14] Safapour, E., Kermanshachi, S., & Pamidimukkala, A. (2021). Post–disaster recovery in urban and rural communities: Challenges and strategies. *International Journal of Disaster Risk Reduction*, *64*, 102535. 10.1016/j.ijdrr.2021.102535

[15] Dewees, S., & Marks, B. (2017). *Twice invisible: Understanding rural Native America*. First Nations Development Institute. https://www.usetinc.org/wp-content/uploads/bvenuti/WWS/2017/May%202017/May%208/Twice%20Invisible%20-%20Research%20Note.pdf

[16] Federal Emergency Management Agency (2017). *Tribal mitigation plan review guide* (Policy FP-306-112-1). U.S. Department of Homeland Security. https://www.fema.gov/sites/default/files/documents/fema-tribal-mitigation-plan-review-guide_12-05-2017.pdf

[17] Schramm, P. J., Rhoades, E. R., Caldwell, R., Klein, J., & Jarris, P. E. (2020). How Indigenous communities are adapting to climate change: Insights from the Climate-Ready Tribes Initiative. *Health Affairs*, *39*(12), 2169–2175. 10.1377/hlthaff.2020.00997 10.1377/hlthaff.2020.00997 PMC8931667 33284701

[18] Davis, C. R., Griffard, M. R., Burton, A., Weinberg, J., Kaneria, K., Smith, M., Sabin, G., & Barnes, T. (2022). A Band-Aid fix to a problem that’s going to be persistent: The influence of social place attachment on rural residents’ perceptions of natural hazard relief efforts. *International Journal of Disaster Risk Reduction*, *67*, 102640. 10.1016/J.IJDRR.2021.102640

[19] Carrasco Cruz, A., Cruz-Souza, F., & González-Calvo, G. (2025). Roots of Rural Youth: A Five-Year Systematic Review of Place Attachment. *Social Sciences*, *14*(9), 554. 10.3390/socsci14090554

[20] Cuthbertson, J., Archer, F., Robertson, A., & Rodriguez-Llanes, J. (2023). A Socio-Health Approach to Improve Local Disaster Resilience and Contain Secondary Crises: A Case Study in an Agricultural Community Exposed to Bushfires in Australia. *Prehospital and disaster medicine*, *38*(1), 3–10. 10.1017/S1049023X22002436 36606323 10.1017/S1049023X22002436 PMC9885428

[21] Federal Emergency Management Agency (2021). *A Guide to Supporting Engagement and Resiliency in Rural Communities.* U.S. Department of Homeland Security. [1] https://www.fema.gov/sites/default/files/documents/fema_rural-guide_jan-2021.pdf

