# Supplementary material for: The Value of Community Building and Social Capital for Rural Communities Recovering from Disaster
Source: J Community Health. 2026 May 24;51(4):687–94. doi: 10.1007/s10900-026-01583-w (PMC13385016; doi:10.1007/s10900-026-01583-w)
Supplement: Supplementary file 1 — Supplementary Material 1. [file 10900_2026_1583_MOESM1_ESM.docx]

*Author Response:*

*Overall, we would like to thank the reviewers for the review and recommendations. We would like to also thank the editor for the opportunity to revise and resubmit the manuscript.*

Reviewer 1:

Comment 1: This is an interesting study. Provide N of 28 in title of table. Delete Quote numbers from table as this comprises anonmity.

*Author Response:*

*Thank you for this thoughtful comment. We appreciate the opportunity to clarify. The number of quotes included in the table does not correspond to the number of participants, as multiple excerpts may come from the same individual. Therefore, including “N=28” could be misleading in this context.*

*To improve clarity, we have revised the table caption to better explain that the excerpts represent illustrative quotes rather than individual participants.*

*Regarding the numbering of quotes, these are used solely for organizational purposes within themes and are not linked to specific individuals. To address concerns about anonymity, we have ensured that no identifying information is conveyed and clarified this in the table.*
